# Taxonomic diversity pattern and composition of fish species in the upper reaches of Ganjiang River, Jiangxi, China

**DOI:** 10.1371/journal.pone.0241762

**Published:** 2020-11-16

**Authors:** Zimo Shi, Jianming Zhang, Haijun Wu, Jing Yang, Maolin Hu

**Affiliations:** 1 School of Life Sciences, Nanchang University, Nanchang, Jiangxi, P. R. China; 2 Ganzhou Fisheries Research Institute, Ganzhou, Jiangxi, P. R. China; 3 Pingxiang Municipal of Fisheries, Pingxiang, Jiangxi, P. R. China; 4 Ministry of Education Key Laboratory of Poyang Lake Environment and Resource Utilization, Nanchang University, Nanchang, Jiangxi, P. R. China; 5 Jiangxi Key Laboratory of Aquatic Animal Resource and Utilization, Nanchang University, Nanchang, Jiangxi, P. R. China; King’s College London, UNITED KINGDOM

## Abstract

Maintaining fish diversity is essential for environmental protection. To characterize the fish composition of the tributaries and mainstream in the upper reaches of the Ganjiang River, we identified seventy-five species of 15 families in 14 sampling sites. These data were analyzed using the inclusion index at the taxonomic level (TINCLi), the importance value index (IVI), the taxonomic diversity indices (Δ^+^ and Λ^+^), cluster analysis and non-metric multidimensional scaling (nMDS). The results showed that the most common and dominant order was the Cypriniformes, represented most frequently by *Hemiculter leucisculus* among the sample sites. Most fishes were omnivorous, mountain stream or settled fish that lay sinking or viscid eggs. Most sites showed a trend of more discrete distribution from high latitude to low latitude. According to the taxonomic diversity indices, the fish taxonomic composition in the upper reaches of Ganjiang River is uneven. The Bray-Curtis resemblance matrix and nMDS showed that the habitats of the Ganjiang River were divided into four areas. The results will provide information needed for freshwater fish resource protection in the upper reaches of the Ganjiang River.

## Introduction

Selection, drift and dispersal usually lead to different number of species and community size in different regions [1]. Drift is random but has important potential effects, it plays an important role in small community, it can override the effects of selection, but in large community with strong selection, all drift effects will be covered by selection. Selection is usually the combined action of abiotic environment and biology [2]. In freshwater fish community, the selection affecting community structure include environmental changes, habitats and anthropogenic influences, etc [3]. At the same time, the direction of community dispersion is also not homogenous. These factors can lead to the heterogeneity of community structure in different regions.

Many fish species can be widely distributed and their distribution is integrally linked to a range of environment factors [4]. These factors provided us a tool for assessing the ecological integrity of rivers [5]. In recent years, fish biodiversity decreased dramatically in China, assessment and conservation of fish resources has become more important [6].

The Ganjiang River is the main and longest river in Jiangxi province, China. To date, scholars have recorded 251 fish species and subspecies in the Ganjiang River basin, 133 of which are thought to be endemic [7]. The river also includes 12 spawning grounds for the four major carps of China, silver carp (*Hypophthalmichthys molitrix*), grass carp (*Ctenopharyngodon idella*), bighead carp (*Hypophthalmichthys nobilis*), and crucian carp (*Carassius carassius*) are the four dominantly consumed freshwater fish in China, accounting for approximately 89% of Chinese freshwater aquaculture [8]. Scholars also have reported a breeding area of *Tenualosa*. *reevesii* in the Ganjiang River [9, 10]. Therefore, the Ganjiang River has an important role in the biodiversity and conservation in the Yangtze River basin.

Anthropogenic influences have greatly promoted the reduction in species and populations in ecosystems worldwide [11–13]. Researches show that the mining and processing of mineral resources in the South and northwest basin of Ganjiang River is the main pollution source of dissolved metals in the Ganjiang River [14, 15]. Addtionally, urban sewage of upstream and downstream of Ganjiang River Basin is the main pollution source affecting the distribution of nitrogen and phosphorus nutrients in the basin [16].

Hydropower (such as the Wan’an Dam) and fishing have posed a great threat to freshwater fish resources in the region and have influenced the distribution and habitat of fishes, resulting in many endemic species becoming endangered [17]. For example, the yield of *T*. *reevesii* in Ganjiang River is declining due to the block of dam [9]. The same condition occured in other river basins after Gezhouba Dam construction, with the proportion of migratory fish decreasing, while common carp and catfish increased perhaps due to the change of connectivity in the Yangtze River [18]. In the Lancang River, within the first year after impounded by the Xiaowan hydropower dam, the fish assemblages changed associated with the introduction of nonnative fish (*Gambusia affinis*) [19] combined with the extirpation of most of the native fish species due to the loss of lotic habitats [20]. Meanwhile, due to the dam construction the associated hydrology gradually changed and the migration of fish species from the lower reaches and egg drift was disrupted. Breeding grounds of four major Chinese carp were also been affected because of reduced water inflow [21]. Historically, two spawning grounds of major carps once existed in the upper reaches of the Ganjiang River [17]; however, the construction of the Wan’an dam has caused those spawning grounds to diminish with only the Laohujiao spawning ground currently remaining [22].

Traditional fisheries are also threatened by overfishing, with the additional pressures of modern harvesting activities [23]. One reason for the decline in fish stocks was overfishing of spawning fish [24]. The impact of illegal fishing is relatively small, but it is practiced widely and causes considerable damage to freshwater fisheries [25]. For example, despite being illegal, several fishing methods (i.e., electricity, traps, and poisons) are still common in China [26]. These methods cause devastating damage to fish stocks, leading to large reductions in fish biodiversity [9, 22, 25].

Sand mining also destroys fish habitats and reproduction grounds [27]. The severe soil erosion and water losses were caused by the synergistic effects of agricultural activity, precipitation, hilly and mountainous terrain, and large-scale construction in Jiangxi Province [11, 28, 29]. Assessing the adverse impact of anthropogenic activities on biodiversity is currently a most pressing task [30]. However, in recent years, no studies have focused on the fish resources and the communities in the upper reaches of the Ganjiang River.

Fish community structure have been shown to be influenced by a number of latitudinally varying environmental factors such as turbidity, temperature, conductivity, and pH. As such, latitudinal gradient of fish diversity are generally a highly predictable spatial pattern [31, 32]. The latitude span of Ganjiang River is large, and the runoff vary greatly, even in the upstream and downstream of the same tributary, with the change of latitude, the water temperature and habitat type are also changing, fish communities may not homogeneous. Under the influence of such complex anthropogenic influences and different environmental factors, freshwater fish communities were expected to be heterogeneous among the tributaries and channel in the upper reaches of Ganjiang River. and the fish community metrics would increase from higher latitude sites than lower latitude sites in this study.

Although, many researchers have investigated fish biodiversity and distribution of in the middle and lower reaches of the Ganjiang River [33], to date there has not been a comprehensive study. Therefore, our main objective was to systematically evaluate fish species diversity of fish in this area, it is important to analyze the spatial distribution and composition of fish in upper reaches of the Ganjiang River comprehensively.

The main aims of the present study were: (1) describe the species composition and taxonomic diversity pattern of the fish community in the upper reaches of the Ganjiang River; and (2) evaluate whether these fish communities exhibit homogenous or latitudinal trends.

## Materials and methods

### Study area

The Ganjiang River is the main and longest river in Jiangxi province, which flows into Poyang Lake and drains into the Yangtze River, it is one of the eight tributaries of the Yangtze River, at a position between 113°30′–116.00°40′E, 27°54'00"–33°4'48"N [7]. The terrain of Jiangxi Province is mainly hilly and mountainous, and the basins and valleys are widespread and slightly plain. The basin control area comprises 82,809 square kilometers. Of these, 81,527 square kilometers are in Jiangxi Province, accounting for 98.45% of the area of the controlled basin. The river system of the Ganjiang River is complicated, the upper reaches charactered by mountainous streams of the river, the water flow of middle and lower reaches river is generally smooth, and the basin is characterized by mid-subtropical humid climate.

The Ganjiang River’s upper reaches are located in Ganzhou City, southern Jiangxi Province, and some tributaries are remitted into the Zhangshui and Gongshui Rivers. The main branches in the upper reaches of the Ganjiang River are: The Shangyujiang River, the Longhua River, and the Zhangjiang River, among which the Shangyujiang and Longhua Rivers have water conservation facilities [34]. The river beach is mainly composed of sand. In the area of the Wan’an reservoir, the Gongjiang River and Zhangjiang Rivers have more artificial disturbance, and the water is deep and flows rapidly. The upstream coast of the Taojiang River has many mining plants. The upper reaches of the Pingjiang River have a large reservoir, and the bottom of the river is mainly sand. The upstream regions of the Meijiang River and Qinjiang River have reservoirs and mountains, with high levels of vegetation. For the Mianshui, Xiangshui, and Lianshui Rivers, the vegetation on both banks of the river is abundant.

### Survey sites

**Fig 1:**
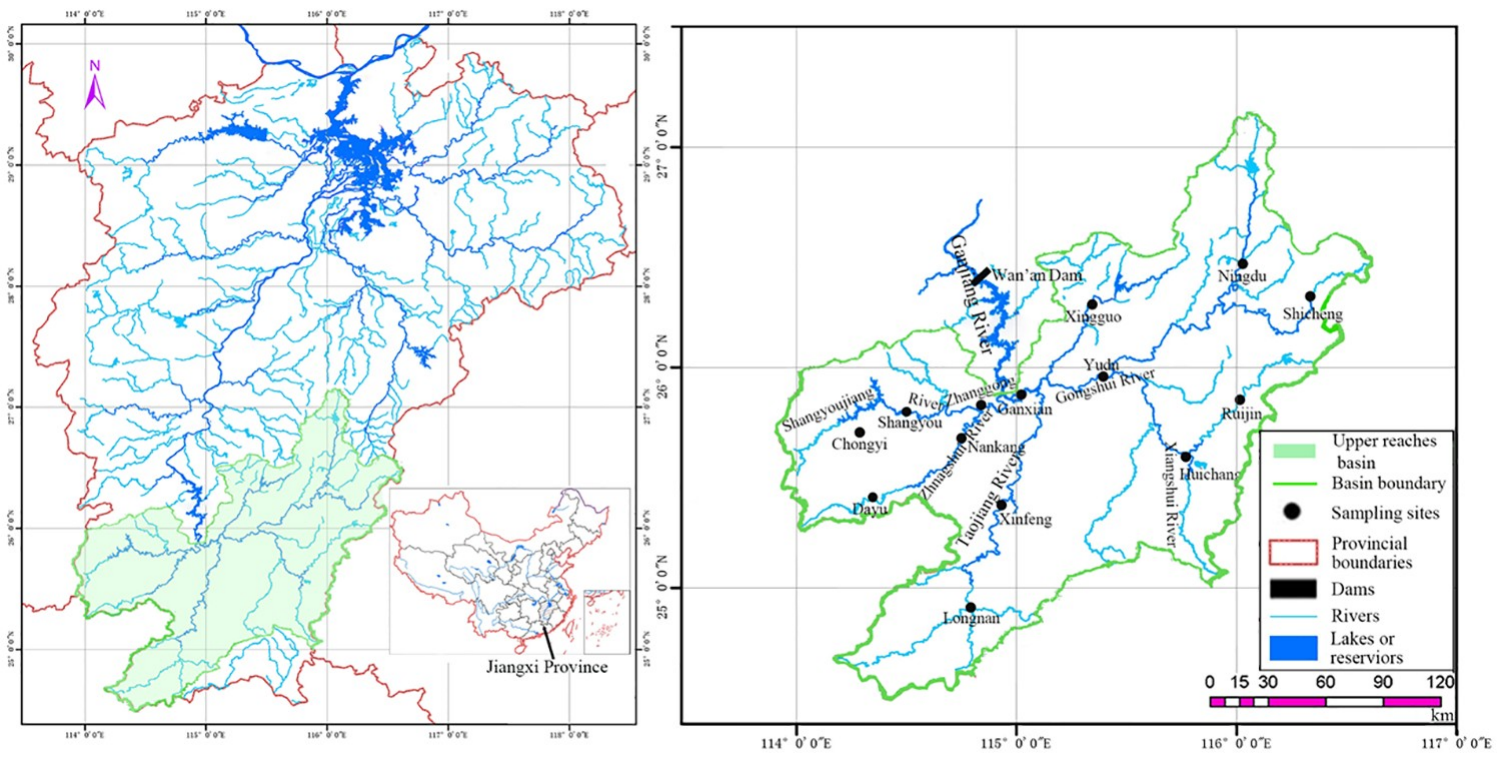
Distribution of sampling sites and dams in upper reaches of the Ganjiang River, Jiangxi Province, China. Reproduced from [33] under a CC BY license, with permission from [SpringerNature], original copyright [2019].

Fish were collected from 14 sites in the upper reaches of the Ganjiang River basin (Fig 1). Site selections were based on habitat types, anthropogenic stressors and accessibility during the sampling period (S1 Table). At each sampling site, we used Garmin GPS map 76Cx to record the GPS position and altitude.

### Sampling method

Samples were taken seasonally from April to May, June to August, September to November, and December to February, from 2016 to 2017. We hired a local fishing boat to catch the fish in each study area where the water depth was more than 1 meter. At each sampling site, a ground cage and a gillnet were used to collect the fish (Table 1).

**Table 1:** The fishing method at each sampling site.

Type	Sizes	Number of nets	Catching time
gillnet	50 × 3 m with five panels (1.5, 3, 4.5, 6 and 7.5-cm bar mesh)	1	20:00–6:00
ground cage	5 m long × 0.5 m × 0.5 m, 5 mm mesh	1	20:00–6:00

As Passive fishing methods, gill net and ground cage have different selectivity to fish. A ground cage has advantages in catching demersal species and smaller mesh size allows for a widely distributed catch of across a range of fish body sizes. Overall, this leads to the catch of small fish species and higher proportion of juvenile fish in the catches. Conversely, gill nets are generally good at catching pelagic fish, with low selectivity for small individuals [35], relatively concentrated body length and relatively few species. Both cages and nets tend to have lower capture efficiency on active species, so some lotic species, small individuals and rare species are generally underrepresented with this sampling methods.

We sorted all individuals into species and counted them on board. Most fish were released after identification; however, individuals that could not be identified were fixed in 10% formalin, and brought back to laboratory for accurate taxonomic verification [9]. Species identifications were carried out according to Zhu [36], Chen [37], Chu et al. [38] and Yue [39]. The division of habitat preferences, feeding habits, trophic ecology, and reproductive behavior across the sampled fishes were according to the Institute of Hydrobiology, Chinese Academy of Sciences.

### Statistics analyses

The Importance Value Index (IVI) was used to analyze dominance of fish species. IVI is a comprehensive index that indicates the functional status of a specific species in the community [40]:

$$\begin{array}{c} \text{IVI}=\text{Fi}(\%)\cdot \text{Pi}(\%)\\ \text{Pi}:\text{Pi}=(\text{Ni}/\text{N})\times 100\%(\text{Relative}\;\text{density})\\ \text{Fi}:\text{Fi}=(\text{Fi}/\text{F})\times 100\%(\text{Occurring}\;\text{frequency}) \end{array}$$

where Fi and Pi are the frequency of the occurrence and relative abundance of species i. We define fish dominance with dominant (>100% by IVI), relatively important (>10% by IVI) and rare (< 10% by IVI).

By analyzing the concentration of species distribution across multiple taxonomic levels (order, family, genus, species) relationships among species can be explained. The higher inclusion index at the taxonomic level (TINCLi) values concentrates more on the distribution of species in the taxonomic hierarchy [41], and it is related to lower taxonomic diversity. The more significant the value, the greater the average quantity of the lower taxonomic level contained in the higher taxonomic level [42].

$${TINCL}_{i\;}\;=\;\frac{1}{N_{i}}\sum_{j=1}^{N_{i}}C_{ki},\;(k\;<\;i)$$

where N_i_ is the quantities of taxonomic level i, C_ki_ is the quantity of the number j in taxonomic level k (order, family, genus, species)

Different to TINCLi, which focuses on the analysis of the concentration of species distribution in each taxonomic level, taxonomic distinctness [43] calculates community structure relationship of species and accesses the complexity of taxonomic trees.

Taxonomic distinctness was proposed by Warwick & Clarke in 1995 [41], which includes indices such as taxonomic diversity (Δ), taxonomic distinctness (Δ*), average taxonomic distinctness (Δ^+^), and variation in taxonomic distinctness (VarTD, Λ^+^). Among them, Δ^+^ and Λ^+^ are not limited by sampling effort, sampling method, or natural variations of habitat type, thus these two indices are significant for the comparative study of different regions, habitats, and historical data, as well as for research into unknown or inconsistent sampling properties. These indices have theoretical advantages over traditional biodiversity indices, which have been proven in microbenthic [44], zooplankton [45], and marine fish [46] communities. However, the taxonomic diversity in freshwater fish has been studied less extensively. The average taxonomic distinctness (Δ^+^) and the variation in taxonomic distinctness (Λ^+^) are used to evaluate the distance between the taxonomy of species and the distance of the hierarchical taxonomic tree [47, 48]. The Δ^+^ value represents the average taxonomic path length between any two species in the community. The higher the value of Δ^+^, the more distant the relationship between species in the community. The Λ^+^ value is the average value deviating from Δ^+^, which reflects the evenness of the distribution of relationships among species of fish [49, 50]. The larger the value of Λ^+^, the more uneven the relationships among species in the community [11, 50]. Δ^+^ refers to the length of the path of a taxonomic level by randomly selecting any two species in the master list (a reference list of fish species collected from the 14 sampling sites), which is a theoretical average value, and the value does not increase with increase number of species. Furthermore, it is determined by the number of species rather than individual quantities, and applied on presence/absence data. Λ^+^ refers to the theoretical average value of the deviation degree from the average taxonomic distinctness, whose value is small only when the number of species is small.

average taxonomic distinctness
  $${\Delta \;}^{+}\;=\;(\sum \sum_{i<j}\;\omega_{ij})/[S(S-1)/2]$$
variation in taxonomic distinctness
  $${\Lambda \;}^{+}\;=\;\sum \sum_{i<j}(\omega ij-\Delta +)2/[S(S-1)/2]$$

where ωij is the distinctness mass given to the path length linking species i and j in the hierarchical classification; S is the total number of fish species in the survey.

In this study, the classification of fish was divided into five levels: class, order, family, genus, and species. The weight of the path length of species belonging to the same phylum, but not the same class; the same class, but not the same order; the same family, but not the same genus; and the same genus, but not the same species were 83.333, 66.667, 50.000, 33.333, and 16.667, respectively [51]. Furthermore, the values of Δ^+^ and Λ^+^ were tested for departure from expectation according to randomization tests. A randomization test with 10,000 random selections was used to detect the expected values of Δ^+^ and Λ^+^ derived from species pool (master list), enabling us to test the significance of departure between the observed and expected values of the two indices. Such plots are described as confidence funnel plots, where degraded sites were assumed to fall below the lower 95% confidence limits, while reference sites should be located within the 95% confidence limits [48]. The calculations of Δ^+^ and Λ^+^ and randomization test were computed using the TAXDTEST procedure in the PRIMER package.

A dataset covering all species collected at each site was then constructed, and similarity analyses were carried out based on logarithmic transformation of the number of each species at each site. Pairwise similarities among sites were then computed to create a similarity coefficient matrix. The hierarchical cluster, furthest-neighbor method with Bray-Curtis similarity was then used for cluster analysis based on the matrix. One-way analysis of similarities (ANOSIM) was used to determine significant differences in under non-metric multidimensional scaling (nMDS) ordination. First, a global R statistic was calculated to determine the significant differences between all groups (analogous to the global F test in analysis of variance (ANOVA)). Significant differences at a global level were then determined using pairwise comparisons between sample groups to test for differences between pairs. In the global test, significance was set at P < 0.05. Additionally, Similarity percentage (SIMPER)were used to determine the contributions of each species to any differences in categorized sites.

All analyses were calculated using SPSS 21.0, CANOCO5.0 and PRIMER (Plymouth Routines In Multivariate Ecological Research) 5.0 [52].

## Results

### Species composition

We collected 29,515 individual fish from the 14 sampling sites throughout this study area, and categorized them into five orders, 15 families, and 75 species (S2 Table). Across all sites, Cypriniformes was the most common order (3 families, 47 species), followed by Perciformes (6 families, 13 species), Siluriformes (4 families, 13 species), while the Synbranchidae and Cyprinodontiformes were represented by only one species, respectively. While the family with highest species richness was Cyprinidae (41 species, 54.66% of the total species collected); followed by Bagridae (nine species, 12%); Cobitidae and Serranidae (five species and 6.67%, respectively); Clariidae, Gobiidae, Anabantidae, and Channidae (two species and 2.67% each). Balitoridae, Siluridae, Amblycipitidae, Synbranchidae, Mastacembelidae, Cichlaidae, and Poeciliidae all had one species each.

*Hemiculter leucisculus* was the most common (71.43% by frequency) and dominant (1772.7% by IVI) species. *Hemiculter leucisculus*, *Xenocypris argentea*, *Squalidus argentatus*, *Hemiculterella sauvagei*, *Carassius auratus*, *Xenocypris microlepis*, *Saurogobio dabryi*, *Xenocypris davidi*, *Saurogobio dumerili*, *Pelteobagrus fulvidraco*, *Cyprinus carpio*, *Hemibarbus labeo*, and *Acrossocheilus parallens* were also common (> 40% by frequency) and dominant (>100% by IVI). Meanwhile, four species were restricted (< 40% by frequency), but were relatively important (>10% by IVI). The other 43 species were rare (< 10% by IVI). (Table 2).

**Table 2:** Occurrence frequency (F), relative abundance (P), index of relative importance (IVI) of fish collected from upper reaches of Ganjiang River.

	F (%)	P (%)	IVI (%)	Ecological type
Cypriniformes				
Cobitidae				
Botiinae				
*Leptobotia taeniaps*	14.29%	0.03%	0.44	MS, O, De, S
Cobitinae				
*Misgurnus anguillicaudatus*	35.71%	0.43%	15.49	MS, O, De, S
*Paramisgurnus dabryanus*	7.14%	0.01%	0.07	MS, O, De, S
*Parabotia fasciata*	7.14%	0.003%	0.02	MS, O, De, Dr
*Cobitis sinensis*	14.29%	0.01%	0.19	MS, O, De, S
Balitoridae				
*Vanmanenia pingchowensis*	14.29%	0.01%	0.15	MS, O, De, S
Cyprinidae				
Danioninae				
*Opsariichthys bidens*	71.43%	0.92%	65.83	MS, C, U, S
*Zacco platypus*	57.14%	1.46%	83.44	MS, C, U, S
*Leuciscinae*				
*Mylopharyngodon piceus*	21.43%	0.02%	0.51	RL, C, L, Dr
*Ctenopharyngodon idellus*	64.29%	0.47%	30.49	RL, H, L, Dr
*Squaliobarbus curriculus*	7.14%	0.01%	0.10	RL, O, U, S
*Elopichthys bambusa*	7.14%	0.00%	0.02	RL, C, L, Dr
Cultcrinae				
*Megalobrama amblycephala*	50.00%	0.20%	10.16	R, H, L, V
*Hemiculter bleekeri warpachowsky*	42.86%	0.98%	41.82	R, O, U, V
*Hemiculter leucisculus*	71.43%	24.82%	1772.71	R, O, U, V
*Hemiculterella sauvagei*	64.29%	4.92%	316.47	R, O, U, V
*Pseudohemiculter hainanensis*	7.14%	0.00%	0.02	R, O, U, V
*Culter alburnus*	42.86%	0.36%	15.25	R, C, U, V
*Cultrichthys erythropterus*	21.43%	0.18%	3.78	R, C, U, V
*Culter mongolicus*	28.57%	0.20%	5.61	R, C, U, V
*Pseudolaubuca engraulis*	14.29%	0.06%	0.92	R, O, U, S
*Megalobrama terminalis*	57.14%	0.17%	9.49	R, H, L, V
*Sinibrama macrops*	50.00%	0.33%	16.26	R, H, L, V
Xcnocyprinae				
*Xenocypris argentea*	50.00%	20.00%	999.83	MS, H, L, Dr
*Xenocypris davidi*	50.00%	3.89%	194.48	R, H, L, V
*Xenocypris microlepis*	64.29%	4.05%	260.50	R, H, L, V
*Distoechodon tumirostris*	21.43%	1.44%	30.78	R, H, L, V
Achcilognathinae				
*Rhodeus ocellatus*	35.71%	0.09%	3.39	R, H, U, Hi
*Paracheilognathus meridianus*	7.14%	0.01%	0.05	R, O, U, Hi
*Acheilognathus tonkinensis*	14.29%	0.01%	0.10	R, O, U, Hi
Gobioninae				
*Abbottina rivularis*	35.71%	0.05%	1.69	MS, O, De, V
*Hemibarbus labeo*	78.57%	2.13%	167.45	MS, C, De, S
*Hemibarbus maculatus*	21.43%	0.41%	8.78	MS, C, De, S
*Pseudorasboraparva*	57.14%	1.07%	60.99	MS, O, L, V
*Sarcocheilichthys kiansiensis*	7.14%	0.03%	0.19	MS, O, L, V
*Saurogobio dumerili*	64.29%	2.75%	176.86	MS, O, De, Dr
*Saurogobio dabryi*	92.86%	2.73%	253.26	MS, O, De, Dr
*Squalidus argentatus*	85.71%	11.04%	946.44	MS, O, L, Dr
*Sarcocheilichthys parvus*	21.43%	0.03%	0.58	MS, O, L, V
*Sarcocheilichthys sinensis*	7.14%	0.01%	0.07	MS, O, L, Dr
*Sarcocheilichthys nigripinnis*	14.29%	0.01%	0.15	MS, O, L, V
Cyprininae				
*Carassius auratus*	100.00%	2.98%	297.81	R, O, De, V
*Cyprinus carpio*	92.86%	1.87%	173.66	R, O, De, V
Hypophthalmichthyinae				
*Aristichthys nobilis*	35.71%	0.12%	4.36	RL, C, U, Dr
*Hypophthalmichthys molitrix*	28.57%	0.08%	2.23	RL, H, U, Dr
Barbinae				
*Acrossocheilus parallens*	85.71%	1.29%	110.36	RL, O, L, S
*Spinibarbus hollandi*	57.14%	0.77%	44.14	MS, O, L, Dr
Siluriformes				
Siluridae				
*Silurus asotus*	50.00%	0.37%	18.30	R, C, L, V
Clariidae				
*Clarias leather*	14.29%	0.04%	0.53	R, C, L, V
*Clarias fuscus*	28.57%	0.12%	3.48	R, C, L, V
*Pdpliobagrus anguillicanuda*	7.14%	0.004%	0.02	MS, O, De, S
Bagridae				
*Hemibagrus macropterus*	35.71%	0.47%	16.94	MS, O, De, S
*Pelteobagrus fulvidraco*	85.71%	2.06%	176.57	MS, O, De, S
*Pelteobagrus eupogon*	14.29%	0.02%	0.29	MS, O, De, S
*Pelteobagrus nitidus*	57.14%	0.46%	26.14	MS, O, De, S
*Pelteobagrus vachelli*	42.86%	0.21%	9.15	MS, O, De, S
*Pseudobagrus albomarginatus*	7.14%	0.01%	0.07	R, C, De, S
*Pseudobagrus tenuis*	28.57%	0.49%	14.04	R, C, De, S
*Pseudobagrus pratti*	14.29%	0.02%	0.24	R, C, De, S
*Leiocassis crassilabris*	14.29%	0.12%	1.69	MS, C, De, S
Synbranchiformes				
Synbranchidae				
*Monopterus albus*	28.57%	0.14%	3.97	R, O, De, Dr
Perciformes				
Serranidae				
*Siniperca chuatsi*	50.00%	0.14%	7.00	R, C, U, Dr
*Siniperca scherzeri*	64.29%	0.26%	16.55	R, C, U, Dr
*Siniperca undulata*	21.43%	0.02%	0.44	R, C, U, Dr
*Siniperca kneriGarman*	28.57%	0.08%	2.32	R, C, U, Dr
*Siniperca roulei*	7.14%	0.03%	0.19	R, C, U, Dr
Gobiidae				
Rhinogobius cliffordpopei	14.29%	0.08%	1.16	MS, C, De, S
Rhinogobius giurinus	50.00%	1.39%	69.63	MS, C, De, S
Anabantidae				
*Macropodus chinensis*	7.14%	0.02%	0.17	MS, O, L, Dr
*Macropodus opercularis*	14.29%	0.06%	0.87	MS, O, L, Dr
Channidae				
*Channa argus*	64.29%	0.22%	14.38	R, C, De, V
*Channa asiatica*	85.71%	0.57%	48.50	R, C, De, V
Mastacembelidae				
*Mastacembelus sinensis*	21.43%	0.14%	3.05	MS, C, De, S
Cichlaidae				
*Oreochromis spp*	7.14%	0.004%	0.02	MS, C, S, L
Cyprinodontiformes				
Poeciliidae				
*Gambusia affinis*	7.14%	0.01%	0.05	R, O, U, V

RL: migratory fish; R: settled fish; MS: Mountain stream fish; H: herbivorous fish; C: Carnivorous fish; O: omnivorous fish; U: pelagic fish; L: Lower fish; De: Demersal fish; S: Sinking egg; Dr: Drifting eggs; V: viscid egg; Hi: Shellfish-like-egg

### Characteristics of the TINCLi

The average number of (family, genus, species), (genus, species), and (species) in its order, family, and genus of the upper reaches of Ganjiang River’s fishes was (3.00, 10.40, 15.00), (3.47, 5) and (1.44). Results from the TINCLi and fish species (Table 3) shows that the highest species richness was observed in Xinfeng, and the lowest species richness was observed in Xingguo. The closest correlation was observed at the genus/order and specifications/order level in Xinfeng, while the furthest relationship was observed at the Genus/Order and Species/Order level in Xingguo. The closest correlation was observed at the Genus/Family, Species/Family, and Species/Genus level in Zhanggong; the furthest correlation was observed at the Genus/Family and Species/Family level in Nankang, and the furthest correlation was observed at the Species/Genus level in Ruijin.

**Table 3:** Comparison about taxonomic distinctness and inclusion index at the taxonomic level (TINCLi)of different sampling sites.

Site	Total Number of Species	Δ^+^	Λ^+^	Family/Order	Genus/Order	Species/Order	Genus/Family	Species/Family	Species/Genus
Longnan	36	56.9	230.4	2.00	5.20	7.20	2.60	3.60	1.38
Xinfeng	41	51.9	274.3	2.00	8.50	10.25	4.25	5.13	1.21
Dayu	29	49.1	276.4	1.50	5.50	7.25	3.67	4.83	1.32
Huichang	36	50.9	267.2	1.75	6.50	9.00	3.71	5.14	1.38
Nankang	18	59.3	159.4	2.25	3.75	4.50	1.67	2.00	1.20
Chongyi	28	51.5	252.6	1.75	5.75	7.00	3.29	4.00	1.22
Shangyou	25	48.7	278.9	1.50	5.50	6.25	3.67	4.17	1.14
Zhanggong	37	45.5	253.7	1.00	5.75	9.25	5.75	9.25	1.61
Ruijin	17	44.1	247.3	1.00	4.00	4.25	4.00	4.25	1.06
Ganxian	21	44.6	256.6	1.25	4.50	5.25	3.60	4.20	1.17
Yudu	26	51.5	225.0	1.75	5.75	6.50	3.29	3.71	1.13
Xingguo	14	46.8	285.3	1.00	3.00	3.50	3.00	3.50	1.17
Shicheng	27	46.1	271.7	1.25	6.00	6.75	4.80	5.40	1.13
Ningdu	35	52.0	283.5	1.50	7.00	8.75	4.67	5.83	1.25

### Taxonomic distinctness of fish assemblage

**Fig 2:**
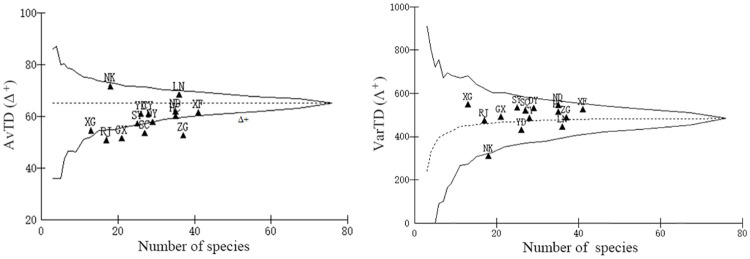
Funnel plots of the average taxonomic distinctness (Δ^+^) and variations in taxonomic distinctness (Λ^+^) in the upper reaches of Ganjiang River.

The results of average taxonomic distinctness(Δ^+^) and variations in taxonomic distinctness (Λ^+^) analysis are shown in Fig 2, and incudes the expected average value of the master list in the upper reaches of Ganjiang River. The 95% confidence funnel ranged from about 10 to 50 species, and the majority of the samples were limited by the confidence funnel for the Δ^+^ values. Most of the Δ^+^ values were lower than the expected average value (64.5), and none of the Δ^+^ values were higher than the limits of the 95% confidence funnel. The Δ^+^ values for the Nankang site (59.3) were higher than those in the other sites. After comparison, the Λ^+^ values were mostly contained in the 95% confidence funnel; only one site (Nankang) fell outside of the limits of the funnel, and showed lower values than the expected Λ^+^ values.

**Fig 3:**
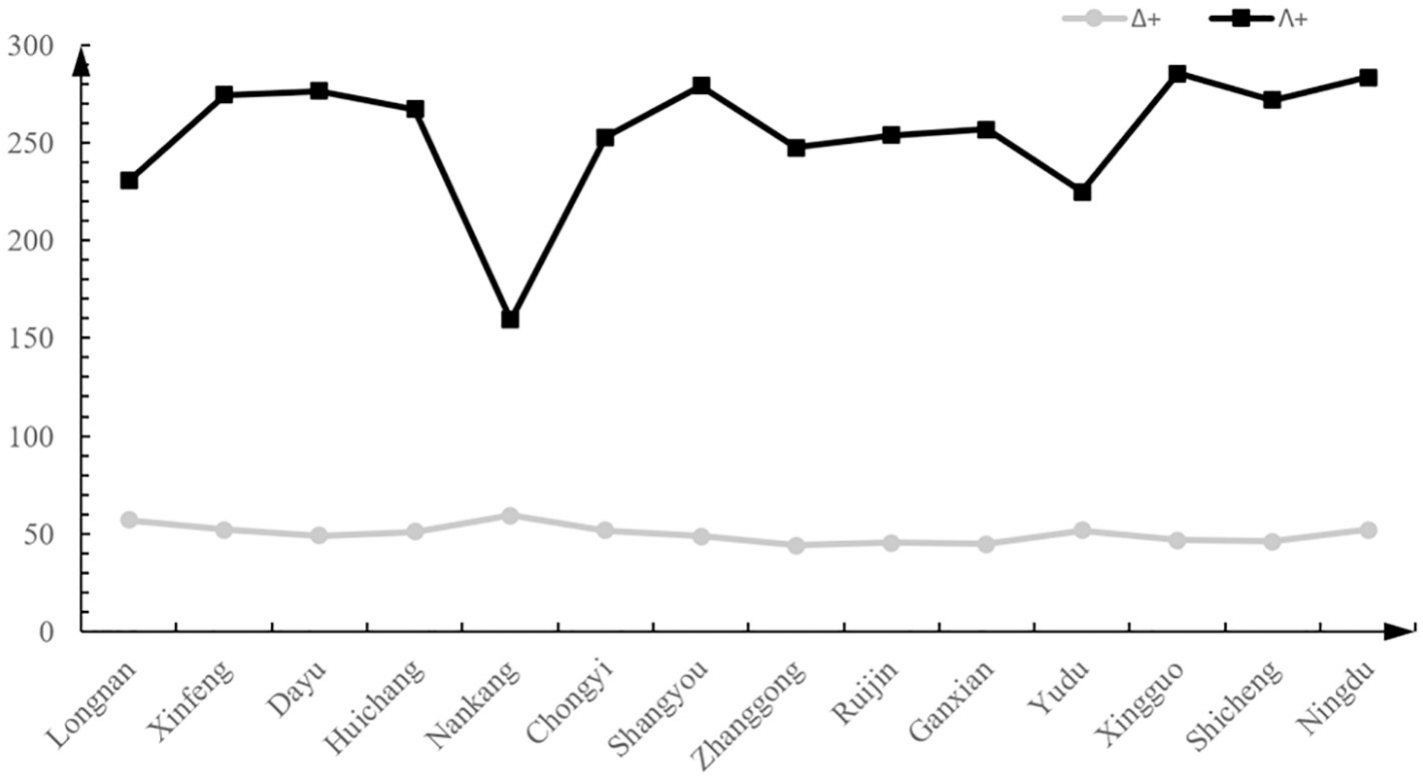
The change trend of average taxonomic distinctness (Δ^+^) and variations in taxonomic distinctness (Λ^+^) from low latitude to high latitude of 14 sites.

Fig 3 shows the changing trend of Δ^+^ values and Λ^+^ values from low latitude to high latitude for the 14 sites (S1 Fig). The results suggested that the Δ^+^ value was lowest in Ganxian and highest in Nankang, while the Λ^+^ value was lowest in Nankang and highest in Ningdu.

**Fig 4:**
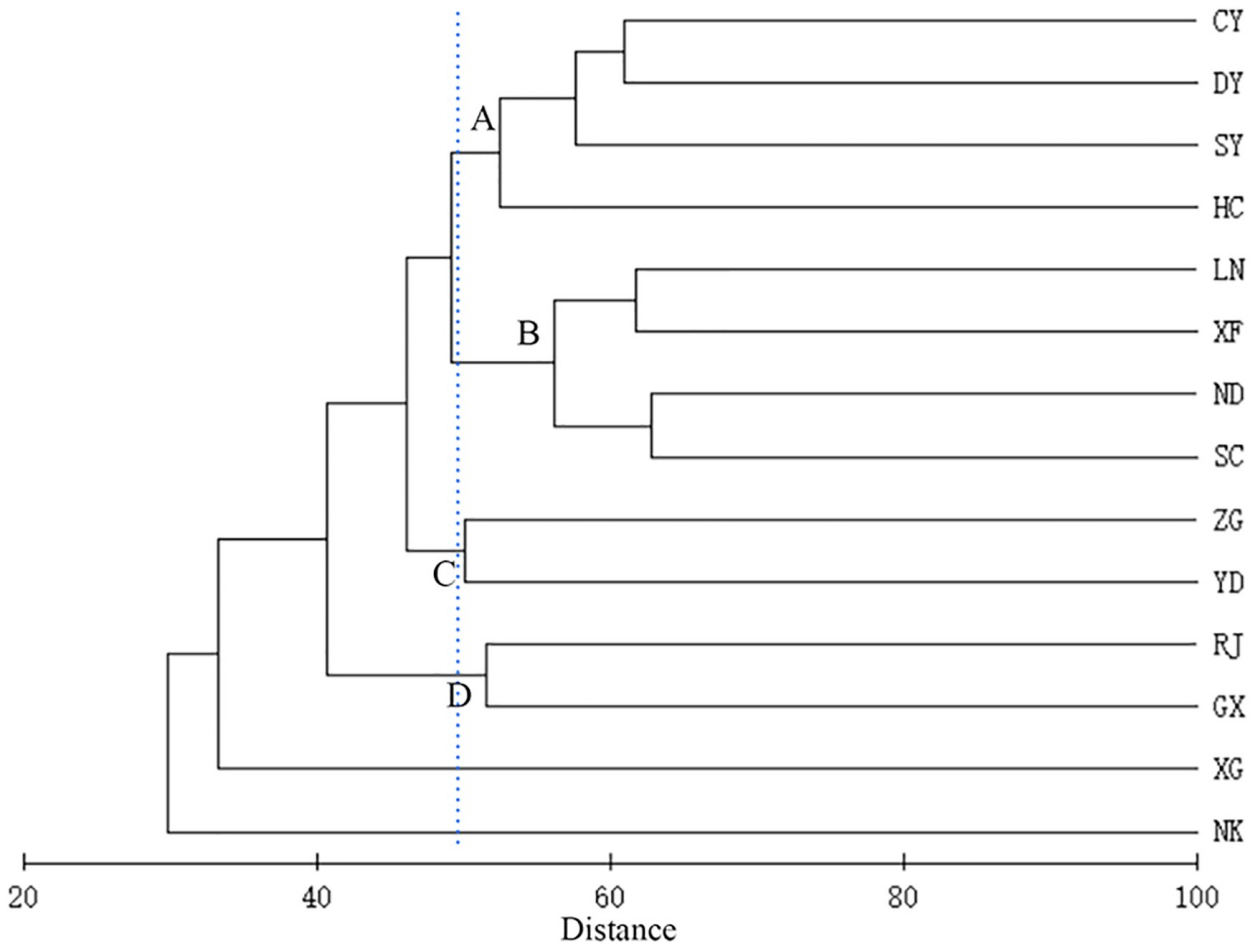
Cluster analyses of the fish species collected at 14 sampling sites in the upper reaches of the Ganjiang River.

Cluster analysis divided the fish species into four significantly different groups (Fig 4). Group A comprised sites Chongyi, Dayu, Shangyou, and Huichang, all located in the Shangyoujiang River, Zhangshui River, and Xiangshui River area, which were the mineral area and agricultural planting area, affected by mining wastewater discharge, slag and soil erosion. Group B covered sites Longnan, Xinfeng, Ningdu, and Shicheng, all located in the Taojiang River and Meijiang River area, which were the concentrated distribution area of iron and coal mine, affected by the waste water from steel smelting. Group C comprised sites Yudu and Zhanggong, which are located at the confluence of the Zhangshui and Gongshui Rivers, and mainly affected by urban sewage. Group D comprised the Ruijin and Ganxian sites, both located in the upper reaches of Gongshui River and at the intersection point of Gongshui River and Ganjiang River, which were affected by the similar pressure of agricultural non-point source pollution.

SIMPER was conducted between fish communities of four groups sampled at 14 sites in the upper reaches of the Ganjiang River (S3 Table). Typifying species within groups and their contributions percentage for fish community structure is showed in this table. Meanwhile seven species (*P*. *dabryanus*, *E*. *bambusa*, *P*. *hainanensis*, *C*. *erythropterus*, *L*. *anguillicanuda*, *S*. *roulei*, and *R*. *cliffordpopei*) were recorded in group A only. Eleven species (*M*. *piceus*, *P*. *meridianus*, *A*. *tonkinensis*, *S*. *nigripinnis*, *P*. *albomarginatus*, *L*. *crassilabris*, *M*. *albus*, *M*. *chinensis*, *M*. *opercularis*, *O*. *spp*, and *G*. *affinis*) were found in these sites only. Five species (*L*. *taeniaps*, *V*. *pingchowensis*, *S*. *curriculus*, *S*. *kiansiensis* and *S*. *sinensis*) appeared only in group C. One species (*P*. *fasciata*) appeared only in group D.

**Fig 5:**
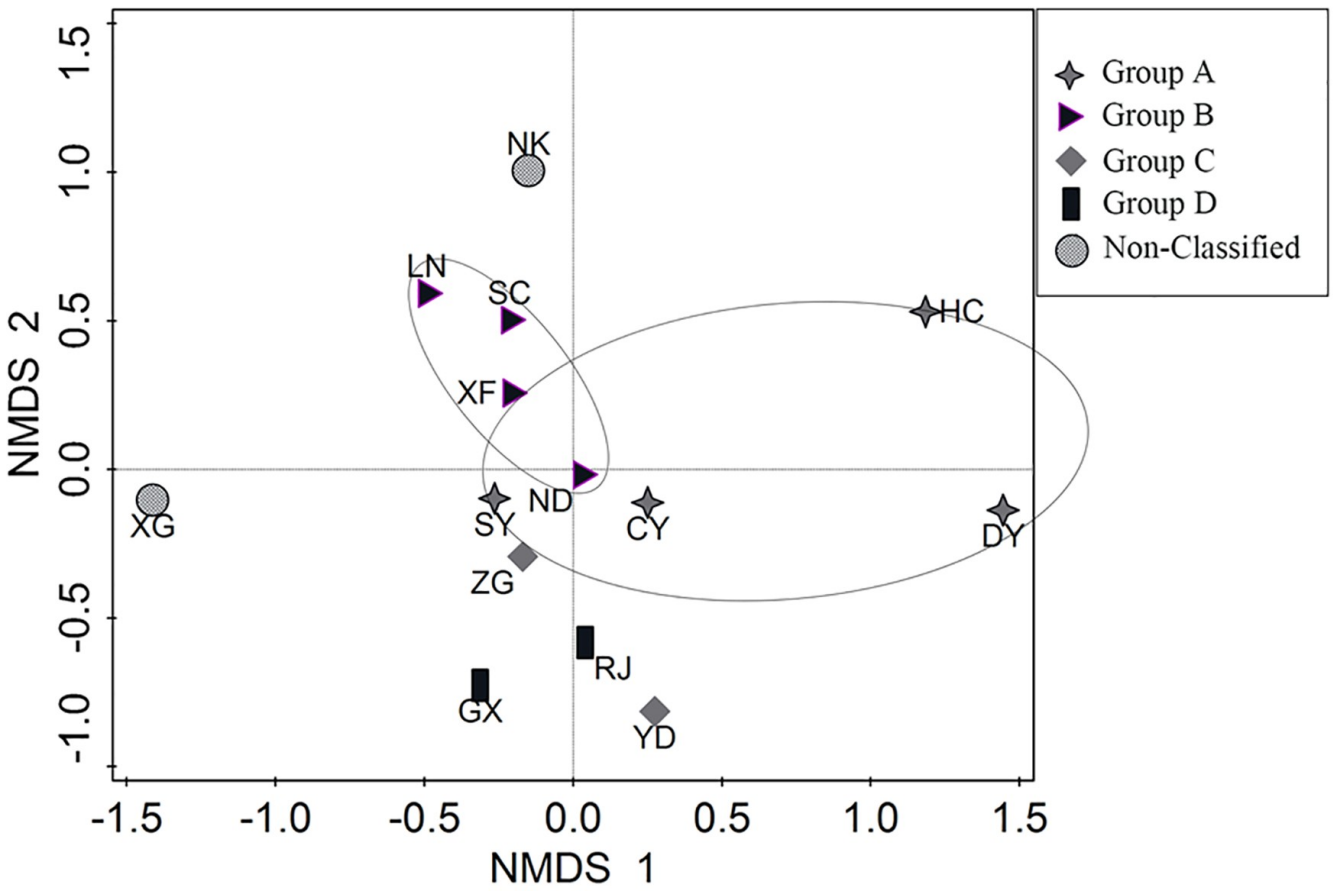
Non-metric multidimensional scaling (nMDS) of the sampling sites based on abundance data.

Non-metric multidimensional scaling ordination also separated the samples into four groups by sampling sites (Fig 5). One-way ANOSIM also revealed a highly significant effect of sampling site (Global test R = 0.797, P = 0.001) based on fish abundance data, represented a significant difference among the four group of those sites.

## Discussion

### Characteristics of fish diversity

Overall, 75 species of fish belonging to 5 orders and 15 families were captured from the upper reaches of the Ganjiang River, represented 29.8% of the total for Jiangxi Province, total number of endemic species represented 22 and 16.5% of the total for Jiangxi Province. Compared with historical fish composition upper reaches of the Ganjiang River of 2009 [22], 13 species didn’t occur in our survey. Additionally, some endemic species such as *Liobagrus styani*, *Ochetobius elongatus*, *Tanichthys albonubes* and *Acipenser sinensis* may be extinct [7]. Cypriniformes accounted for a relatively highest proportion, and Poeciliidae contributed the lowest proportion of species. This result corresponds to the distribution of fish in the Yangtze River and the Ganjiang River as a whole.

Anthropogenic activities can display a great influence in some of the aspects in richness and compositon [53] and contribute to significant differences in the expected patterns along a basin. For example, dam constructions in the lower reach of Ganjiang River and Poyang Lake will cut off the connectivity among the Ganjiang River, Poyang Lake and the Yangtze River in the future [54]. Due to the barrier of dam constructions, the environmental factors such as water depth, pH and dissolved oxygen changed [55], it is harmful to the reproduction of fish with drifting eggs(i.e., *H*. *molitrix*, *C*. *Idella*, *H*. *nobilis* and *C*. *carassius*) and the development of fish with viscid eggs(i.e., *C*. *auratus*, *C*. *carpio*) [17], the fish community in the upper reaches of Ganjiang River may change in future.

According to the TINCL_i_ value and the species number for the 14 sampling sites, we observed the highest species richness in Xinfeng, the site is located in the western of Jinpenshan Nature Reserve with low environmental stress. Whereas Xingguo displayed the lowest species richness, the site is located in the Concentrated distribution area of tungsten mine under high environmental stress. Furthermore, a higher TINCLi value was observed the in Xinfeng and Zhanggong sites. This indicated that the diversity of fish species in Xinfeng and Zhanggong were comparatively centralized, which is related to the high environmental stress caused by human activities. The values for the other sites showed a trend of more discrete distribution from high latitude to low latitude. IVI analysis showed that most species were omnivorous, mountain stream or settled fish, which lay sinking or viscid eggs. Common species (i.e., *S*. *argentatus*) are well adapted to living in small streams with freshwater and a relatively high dissolved oxygen concentration.

Our results showed that the Δ^+^ value tended to diminish from the tributary to the mainstream, and from low latitude to high latitude (S1 Fig). This result is in line with the expectation of the diversity shows a negative relationship with latitudinal location across the basin. This trend is contrary to the taxonomic distinctness indices of fish at different latitudes among the China Seas: “South low north high” [56]. Meanwhile, Longnan and Nankang showed higher Δ^+^ values, it means that the fish diversity of these two points is relatively high. The Δ^+^ values fell very close to the lower limit of the funnel for 12 sites, and were far below for Ruijin, Ganxian, Shicheng, and Zhanggong. Thus, the fish population structure in these areas is relatively unstable. It may be due to overfishing and habitat destruction, which have greatly affected the composition of fish species and their taxonomic diversity [57]. Detrimental interactions with hatchery-reared fish also has adverse effects on local freshwater fish stocks [58]. At the same time, there are many mineral mining areas in the upper reaches of Ganjiang River, and the water pollution caused by them deserves attention. These factors posed a serious threat to the community structure of fish in the upper reaches of Ganjiang River. Once the disturbance intensifies, these unstable population structures may be further damaged. However, limited by the lack of physical and chemical data in the same period as a reference, more research on the relationship between taxonomic diversity and environmental factor is needed.

The Λ^+^ analyses revealed that except for Nankang, most sites fell ithin the limit of the funnel. Furthermore, most sites were above the average value of Δ^+^, which suggested that the fish taxonomic composition in the upper reaches of Ganjiang River is heterogeneously distributed. Meanwhile, there is significant departure among the values for the 14 sites, which reflects the prominent difference in phylogenetic taxonomic trees among sub-watershed populations [45].

The results of the cluster analysis and nMDS showed that the habitats in the upper reaches of Ganjiang River could be divided into four groups, which included different tributary rivers and habitats.

### Conservation recommendations

The status of freshwater fish stocks needs to be evaluated comprehensively, and the data should be updated immediately to help the government to determine appropriate strategies of fish conservation.

Habitat protection is also very important. It is imperative to establish reserve areas to protect freshwater fish [59, 60].However, no protected area of freshwater fish had been established in the upper reaches of the Ganjiang River. At present, fish resources are still affected by human activities such as mineral exploitation. Therefore, freshwater protected areas need to be developed immediately [61, 62]. According to the IVI in this study, mountain stream fish species (i.e., *S*. *sinensis*) need to be given attention, and compared with the study in 2009 [22], 13 species have disappeared, research on the life stories of those species is needed. According to the index of taxonomic distinctness and cluster analysis, nature reserves should be established in Longnan and Nankang of Zhangshui River and Taojaing River, Δ^+^ values of those areas are high and the fish communities are relatively complete. At the same time, attention should also be paid to the restoration of water quality in areas with low Δ^+^ values such as Ruijin, Ganxian, Shicheng and Zanggong in tributaries of eastern and central parts in the region.

According to the fishery laws issued by Jiangxi Province in 2019, fishing is prohibited in Xinyu, Shicheng, Ningdu, Xingguo, Shangyou, and Ganxian starting in 2020. This policy will play an essential role in the restoring populations of fish stocks of the Ganjiang River. Unfortunately, many locals are still using electro-fishing or other methods fishing, thus the enforcement of the law will needs to be stricter [63], and further improved protection measures need to be carried out as soon as possible [64].

### Ethics statement

The study was approved by the Institutional Animal Care and Use Committee (IACUC) of Nanchang University, Jiangxi, China. All necessary permits were obtained for the described field studies from the IACUC of Nanchang University and the Yangtze River Fishery Administration of China. The handling of fish was also conducted in accordance with the guidelines on the care and use of animals for scientific purposes set by IACUC of Nanchang University, Jiangxi, China.

## Supporting information

S1 Fig Linear regression between Δ+ values and the latitude of 14 sampling sites. Significant low negative correlation between Δ+ values and latitude (P<0.05).
(TIF)

S1 Table Habitat characteristics of the 14 sampling sections in the upper reaches of the Ganjiang River. (DOCX)

S2 Table Fish composition and distribution in the upper reaches of the Ganjiang River. (DOCX)

S3 Table Typifying species within station groups and their contributions percentage for fish community structure (%). (DOCX)
