# Supplementary figures and images for: Taxonomic diversity pattern and composition of fish species in the upper reaches of Ganjiang River, Jiangxi, China
Source: PLoS One. 2020 Nov 16;15(11):e0241762. doi: 10.1371/journal.pone.0241762 (PMC7668606; doi:10.1371/journal.pone.0241762)

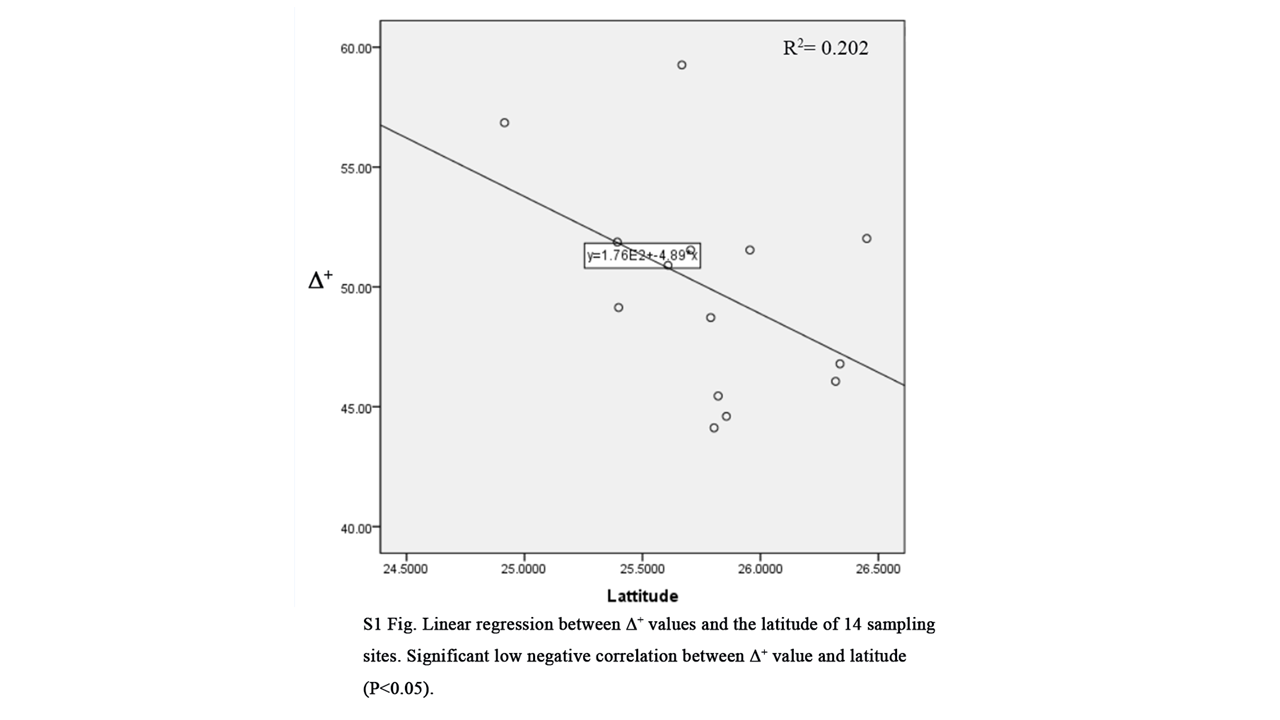

Supplement: S1 Fig — Significant low negative correlation between Δ+ values and latitude (P<0.05). (TIF) [file pone.0241762.s001.tif]
