# Supplementary material for: Taxonomic diversity pattern and composition of fish species in the upper reaches of Ganjiang River, Jiangxi, China
Source: PLoS One. 2020 Nov 16;15(11):e0241762. doi: 10.1371/journal.pone.0241762 (PMC7668606; doi:10.1371/journal.pone.0241762)
Supplement: S1 Table — (DOCX) [file pone.0241762.s002.docx]

**S1Table.** Habitat characteristics of the 14 sampling sections in the upper reaches of the Ganjiang River

| Sampling site | Latitude | Longitude | Stream basin | Habitat descriptions |
| --- | --- | --- | --- | --- |
| SY: Shangyou | 25°47'21"N | 114°33'49"E | Shangyoujiang River | abundant vegetation, rapid water flow, mineral area and agricultural planting area |
| DY: Dayu | 25°23'56"N | 114°21'45"E | Zhangshui River | abundant vegetation, rapid water flow, mineral area and agricultural planting area |
| XF: Xinfeng | 25°23'36"N | 114°56'36"E | Taojiang River | abundant vegetation, rapid water flow, mineral area |
| GX: Ganxian | 25°51'20"N | 115°1'56"E | Gongshui River | sparse vegetation, slow water flow, urban area |
| RJ: Ruijin | 25°48'12"N | 115°59'27"E | Gongshui River | sparse vegetation, slow water flow, agricultural planting area |
| SC: Shicheng | 26°19'6"N | 116°20'24"E | Meijiang River | abundant vegetation, rapid water flow, mineral area |
| HC: Huichang | 25°36'30"N | 115°47'33"E | Xiangshui River | sparse vegetation, slow water flow, mineral area and agricultural planting area |
| XG: Xingguo | 26°20'14"N | 115°22'49"E | Gongshui River | abundant vegetation, rapid water flow, agricultural planting area |
| CY: Chongyi | 25°42'15"N | 114°19'5"E | Shangyoujiang River | abundant vegetation, rapid water flow, mineral area and agricultural planting area |
| NK: Nankang | 25°40'1"N | 114°46'29"E | Zhangshui River | sparse vegetation, slow water flow, mineral area and agricultural planting area |
| LN: Longnan | 24°54'55"N | 114°47'3"E | Taojiang River | abundant vegetation, slow water flow, mineral area |
| YD: Yudu | 25°57'19"N | 115°24'56"E | Gongshui River | sparse vegetation, slow water flow, urban area |
| ND: Ningdu | 26°27'2"N | 116°1'21.9"E | Meijiang River | abundant vegetation, rapid water flow, mineral area |
| ZG: Zhanggong | 25°49'15"N | 114°53'22.8"E | Zhangshui River | sparse vegetation, slow water flow, urban area |
