# Supplementary material for: Taxonomic diversity pattern and composition of fish species in the upper reaches of Ganjiang River, Jiangxi, China
Source: PLoS One. 2020 Nov 16;15(11):e0241762. doi: 10.1371/journal.pone.0241762 (PMC7668606; doi:10.1371/journal.pone.0241762)
Supplement: S2 Table — (DOCX) [file pone.0241762.s003.docx]

**S2Table.** Fish composition and distribution in the upper reaches of the Ganjiang River

| Species | LN | XF | DY | HC | NK | CY | SY | ZG | RJ | GX | YD | XG | SC | ND |
| --- | --- | --- | --- | --- | --- | --- | --- | --- | --- | --- | --- | --- | --- | --- |
| **Cobitidae** |  |  |  |  |  |  |  |  |  |  |  |  |  |  |
| *Leptobotia taeniaps* |  |  |  |  |  |  |  | + |  |  | + |  |  |  |
| *Misgurnus anguillicaudatus* |  |  | + | + | + | + |  | + |  |  |  |  |  |  |
| *Paramisgurnus dabryanus* |  |  |  |  |  | + |  |  |  |  |  |  |  |  |
| *Parabotia fasciata* |  |  |  |  |  |  |  |  |  |  | + |  |  |  |
| *Cobitissinensis* |  |  |  | + |  |  |  |  |  |  | + |  |  |  |
| **Balitoridae** |  |  |  |  |  |  |  |  |  |  |  |  |  |  |
| *Vanmanenia pingchowensis* |  |  |  |  | + |  |  |  |  |  | + |  |  |  |
| **Cyprinidae** |  |  |  |  |  |  |  |  |  |  |  |  |  |  |
| *Opsariichthys bidens* | + | + |  | + |  | + | + | + | + | + |  |  | + | + |
| *Zacco platypus* | + | + |  | + |  |  | + |  | + | + |  |  | + | + |
| *Mylopharyngodon piceus* |  | + |  |  | + |  |  |  |  |  |  |  | + |  |
| *Ctenopharyngodon idellus* |  | + | + | + |  | + | + | + |  | + |  |  | + | + |
| *Squaliobarbus curriculus* |  |  |  |  |  |  |  | + |  |  |  |  |  |  |
| *Elopichthys bambusa* |  |  |  |  |  | + |  |  |  |  |  |  |  |  |
| *Megalobrama amblycephala* |  | + | + | + |  | + | + | + |  |  |  |  | + |  |
| *Hemiculter bleekeri warpachowsky* |  | + | + | + |  | + | + |  |  |  |  |  |  | + |
| *Hemiculter leucisculus* |  |  | + | + |  | + | + | + | + | + | + | + |  | + |
| *Hemiculterella sauvagei* |  | + | + | + |  | + |  | + | + | + |  |  | + | + |
| *Pseudohemiculter hainanensis* |  |  | + |  |  |  |  |  |  |  |  |  |  |  |
| *Culter alburnus* |  |  | + | + |  |  |  | + | + |  |  |  | + | + |
| *Cultrichthys erythropterus* |  |  |  | + |  |  |  | + |  |  |  |  |  | + |
| *Culter mongolicus* |  |  |  | + |  |  | + | + |  | + |  |  |  |  |
| *Pseudolaubuca engraulis* |  |  |  |  |  |  | + |  |  | + |  |  |  |  |
| *Megalobrama terminalis* | + | + | + |  |  |  | + | + |  |  | + |  | + | + |
| *Sinibrama macrops* | + | + | + |  |  |  |  | + |  |  | + |  | + | + |
| *Xenocypris argentea* |  | + | + | + |  | + |  |  | + |  |  | + | + |  |
| *Xenocypris davidi* |  | + | + |  |  | + | + | + |  | + | + |  |  |  |
| *Xenocypris microlepis* | + | + |  | + | + |  |  | + |  | + |  | + | + | + |
| *Distoechodon tumirostris* |  |  |  |  |  |  |  |  |  | + |  |  | + | + |
| *Rhodeus ocellatus* |  |  |  | + | + |  | + |  | + |  |  |  | + |  |
| *Paracheilognathus meridianus* |  | + |  |  |  |  |  |  |  |  |  |  |  |  |
| *Acheilognathus tonkinensis* | + | + |  |  |  |  |  |  |  |  |  |  |  |  |
| *Abbottina rivularis* |  | + | + |  | + |  |  | + |  |  | + |  |  |  |
| *Hemibarbus labeo* | + | + | + | + |  |  | + | + |  | + | + | + | + | + |
| *Hemibarbus maculatus* | + |  |  |  |  |  |  | + |  | + |  |  |  |  |
| *Pseudorasboraparva* | + | + | + | + |  | + |  | + | + |  | + |  |  |  |
| *Sarcocheilichthys kiansiensis* |  |  |  |  |  |  |  | + |  |  |  |  |  |  |
| *Saurogobio dumerili* | + |  | + | + |  |  |  | + | + | + | + | + |  | + |
| *Saurogobio dabryi* | + | + | + | + |  | + | + | + | + | + | + | + | + | + |
| *Squalidus argentatus* |  | + | + | + |  | + | + | + | + | + | + | + | + | + |
| *Sarcocheilichthys parvus* |  |  |  |  |  |  |  | + |  |  | + |  |  | + |
| *Sarcocheilichthys sinensis* |  |  |  |  |  |  |  | + |  |  |  |  |  |  |
| *Sarcocheilichthys nigripinnis* |  | + |  |  |  |  |  |  |  |  |  |  | + |  |
| *Carassius auratus* | + | + | + | + | + | + | + | + | + | + | + | + | + | + |
| *Cyprinus carpio* | + | + | + | + | + | + | + | + | + |  | + | + | + | + |
| *Aristichthys nobilis* | + | + |  |  |  | + |  | + |  |  |  |  |  | + |
| *Hypophthalmichthys molitrix* | + | + |  |  |  | + |  | + |  |  |  |  |  |  |
| *Acrossocheilus parallens* | + | + | + | + |  | + | + | + | + |  | + | + | + | + |
| *Spinibarbus hollandi* | + | + |  | + |  |  | + |  |  | + | + |  | + | + |
| *Silurus asotus* | + | + |  |  |  | + | + |  |  |  |  | + | + | + |
| **Clariidae** |  |  |  |  |  |  |  |  |  |  |  |  |  |  |
| *Clarias leather* | + |  |  | + |  |  |  |  |  |  |  |  |  |  |
| *Clarias fuscus* | + |  |  | + | + | + |  |  |  |  |  |  |  |  |
| **Amblycipitidae** |  |  |  |  |  |  |  |  |  |  |  |  |  |  |
| *Liobagrus anguillicanuda* |  |  |  |  |  | + |  |  |  |  |  |  |  |  |
| **Bagridae** |  |  |  |  |  |  |  |  |  |  |  |  |  |  |
| *Hemibagrus macropterus* | + | + |  |  | + |  |  | + |  |  |  |  |  | + |
| *Pelteobagrus fulvidraco* | + | + | + | + | + | + | + | + | + |  | + |  | + | + |
| *Pelteobagrus eupogon* | + |  |  |  |  |  |  | + |  |  |  |  |  |  |
| *Pelteobagrus nitidus* | + | + | + |  |  | + |  | + |  |  | + |  | + | + |
| *Pelteobagrus vachelli* | + |  | + |  |  | + |  |  |  | + |  |  | + | + |
| *Pseudobagrus albomarginatus* |  |  |  |  |  |  |  |  |  |  |  |  |  | + |
| *Pseudobagrus tenuis* |  | + |  |  |  |  |  |  |  |  | + | + |  | + |
| *Pseudobagrus pratti* |  | + |  |  |  | + |  |  |  |  |  |  |  |  |
| *Leiocassis crassilabris* | + | + |  |  |  |  |  |  |  |  |  |  |  |  |
| **Synbranchidae** |  |  |  |  |  |  |  |  |  |  |  |  |  |  |
| *Monopterus albus* | + |  |  |  | + |  |  |  |  |  |  |  | + | + |
| **Serranidae** |  |  |  |  |  |  |  |  |  |  |  |  |  |  |
| *Siniperca chuatsi* | + | + |  | + | + |  | + | + |  |  | + |  |  |  |
| *Siniperca scherzeri* | + | + | + | + | + |  | + | + |  |  |  | + |  | + |
| *Siniperca undulata* |  | + |  | + |  |  |  |  |  |  |  |  |  | + |
| *Siniperca kneriGarman* | + |  | + |  |  |  |  | + |  |  |  |  |  | + |
| *Siniperca roulei* |  |  |  | + |  |  |  |  |  |  |  |  |  |  |
| **Gobiidae** |  |  |  |  |  |  |  |  |  |  |  |  |  |  |
| *Rhinogobius cliffordpopei* |  |  |  | + | + |  |  |  |  |  |  |  |  |  |
| *Rhinogobius giurinus* |  |  | + | + | + |  | + |  | + | + | + |  |  |  |
| **Anabantidae** |  |  |  |  |  |  |  |  |  |  |  |  |  |  |
| *Macropodus chinensis* | + |  |  |  |  |  |  |  |  |  |  |  |  |  |
| *Macropodus opercularis* | + | + |  |  |  |  |  |  |  |  |  |  |  |  |
| **Channidae** |  |  |  |  |  |  |  |  |  |  |  |  |  |  |
| *Channa argus* | + | + | + | + | + | + | + |  |  |  | + |  |  | + |
| *Channa asiatica* | + | + | + | + | + | + | + |  | + | + | + |  | + | + |
| **Mastacembelidae** |  |  |  |  |  |  |  |  |  |  |  |  |  |  |
| *Mastacembelus sinensis* | + | + |  |  |  |  |  |  |  | + |  |  |  |  |
| **Cichlaidae** |  |  |  |  |  |  |  |  |  |  |  |  |  |  |
| *Oreochromis spp* |  | + |  |  |  |  |  |  |  |  |  |  |  |  |
| **Poeciliidae** |  |  |  |  |  |  |  |  |  |  |  |  |  |  |
| *Gambusia affinis* | + |  |  |  |  |  |  |  |  |  |  |  |  |  |
