# Supplementary material for: Taxonomic diversity pattern and composition of fish species in the upper reaches of Ganjiang River, Jiangxi, China
Source: PLoS One. 2020 Nov 16;15(11):e0241762. doi: 10.1371/journal.pone.0241762 (PMC7668606; doi:10.1371/journal.pone.0241762)
Supplement: S3 Table — (DOCX) [file pone.0241762.s004.docx]

**S3Table** Typifying species within station groups and their contributions percentage for fish community structure (%)

| Species | Group A | | Group B | | Group C | | Group D | |
| --- | --- | --- | --- | --- | --- | --- | --- | --- |
|  | Av.Sim | Con | Av.Sim | Con | Av.Sim | Con | Av.Sim | Con |
| *Hemiculter leucisculus* | 3.35 | 17.13 |  |  | 12.01 | 34.48 |  |  |
| *Hemiculterella sauvagei* | 2.64 | 13.47 |  |  |  |  | 2.76 | 7.93 |
| *Squalidus argentatus* | 2.28 | 11.64 |  |  | 41.44 | 85.18 |  |  |
| *Xenocypris argentea* | 2.25 | 11.51 |  |  |  |  |  |  |
| *Carassius auratus* | 1.72 | 8.82 | 2.77 | 8.07 |  |  |  |  |
| *Xenocypris davidi* | 7.72 | 8.80 |  |  |  |  |  |  |
| *Pelteobagrus fulvidraco* | 1.22 | 6.21 | 4.67 | 13.62 |  |  |  |  |
| *Xenocypris microlepis* |  |  | 5.38 | 15.66 |  |  |  |  |
| *Hemibarbus labeo* |  |  | 3.60 | 10.48 |  |  |  |  |
| *Cyprinus carpio* |  |  | 2.37 | 6.90 |  |  |  |  |
| *Zacco platypus* |  |  | 2.14 | 6.23 |  |  | 6.13 | 17.59 |
| *Spinibarbus hollandi* |  |  | 1.94 | 5.65 |  |  |  |  |
| *Saurogobio dabryi* |  |  |  |  |  |  | 7.81 | 22.41 |
| *Opsariichthys bidens* |  |  |  |  |  |  | 4.56 | 13.10 |

Av.Sim: Average similarity; Con: Rate of contribution. The same as below
